# Understanding the regulatory-procurement interface for medicines in Africa via publicly available information on standards, implementation, and enforcement in five countries

**DOI:** 10.1080/20523211.2024.2436898

**Published:** 2025-01-09

**Authors:** Jillian C. Kohler, Mariangela Castro-Arteaga, Saher Panjwani, David Mukanga, Murray M. Lumpkin, Bonface Fundafunda, Anthony B. Kapeta, Chimwemwe Chamdimba, Anna S. Y. Wong, Kristin N. Harper, Charles Preston

**Affiliations:** a Leslie Dan Faculty of Pharmacy, University of Toronto, Toronto, Canada; b Dalla Lana School of Public Health, University of Toronto, Toronto, Canada; c Munk School of Global Affairs & Public Policy, University of Toronto, Toronto, Canada; d Department of Philosophy, Rotman Institute of Philosophy, Western University, London, Canada; e Bill & Melinda Gates Foundation, Seattle, WA, USA; f Africa Resource Centre, Cape Town, South Africa; g AUDA-NEPAD, Midrand, South Africa; h Faculty of Law, University of Toronto, Toronto, Canada; i Harper Health & Science Communications LLC, Seattle, WA, USA

**Keywords:** National procurement agency, national regulatory authority, substandard and falsified medicines, regulatory-procurement interface, *Pharmaceutical System Transparency and Accountability Assessment Tool*, access to medicines, medicines quality, sub-Saharan Africa

## Abstract

**Background:**

Substandard and falsified medicines in Africa are a major public health concern. Access to quality medical products in African countries is governed in large part by two major entities at the national level: the regulatory authority and the procurement agency. The importance of national regulators in ensuring quality medical products is well known. The interplay between the national regulator and the national procurement agency also has a significant impact on access to quality medicines but is less understood. This study’s aim was to characterise the regulatory-procurement interface – the intersection of decision-making in these two spheres – using publicly available data from five African countries.

**Methods:**

For the five target countries, we adapted criteria from WHO’s 2018 *Pharmaceutical System Transparency and Accountability Assessment Tool* to identify key national policies and practices around the nexus of medicines regulation and procurement.

**Results:**

Though legal and policy frameworks enabling best practices in procurement were often in place, implementation and enforcement of these practices appear to be key areas for strengthening. In addition, we documented a lack of publicly available information related to the role that quality plays in selecting medical products. Finally, none of the five countries have publicly published the results of their selection decisions with key product details, making it difficult to assess whether basic quality standards are being met.

**Conclusion:**

Based on these findings, one of the most important next steps for improving the effectiveness and transparency of national procurement is for procurement agencies to publish detailed quality selection criteria and an up-to-date list of the medical products they have purchased, with key product information. We hope these findings can help inform the conversation about implementing and enforcing best practices at the regulatory-procurement interface, with the goal of improving access to quality versions of medical products in Africa and globally.

## Background

Ensuring access to safe, efficacious quality medicines is central to Sustainable Development Goal (SDG) 3, to ‘ensure healthy lives and promote well-being for all’ (United Nations, 2024), and aligns with the African Union’s Agenda 2063 goal of healthy and well-nourished citizens (African Union, 2013). It is also a critical component of the health systems strengthening necessary to support universal health coverage, a strategic priority for the World Health Organization (WHO) and many countries around the world (Mhazo & Maponga, 2022; Ozawa et al., 2018; WHO, 2024). Unfortunately, lack of access to quality versions of medical products continues to present a major challenge to achieving these goals. Substandard and falsified (SF) versions of medical products are an important contributor to this problem (WHO, 2018b).

SF medicines in Africa are a long-standing public health concern. The worldwide prevalence of SF medicines is estimated to be about 10% (WHO, 2018b). However, one study found 35% of antimalarial drugs in sub-Saharan Africa failed chemical analysis (Nayyar et al., 2012). A study of oxytocin, which plays a central role in the induction of labour and prevention of postpartum hemorrhage, found roughly 40% of samples collected in Africa failed quality assessment (Ammerdorffer et al., 2022). Consistent with these findings, WHO has shared that 42% of global reports of SF drugs come from Africa (WHO, 2017). There is no starker life-and-death example of SF drugs’ effects than the recent cluster of poisonings from tainted cough syrup including diethylene glycol that recently killed 66 children in Gambia (Bastani et al., 2023).

Access to quality versions of medical products in most African countries is typically governed by two major entities at the national level: the national regulatory authority (NRA) and the national procurement agency. To date, global and regional efforts to improve access to quality versions of medical products have often focused on strengthening NRAs (Guzman et al., 2020; O'Brien et al., 2021; Preston et al., 2020). National procurement agencies also play a critical role in ensuring access to quality versions of medical products, though. Ideally, a country’s regulatory and procurement agencies would work together to ensure citizens receive access to products that meet international standards for safety, efficacy, and manufacturing quality. Unfortunately, it can be challenging for regulatory and procurement agencies in low- and middle-income countries (LMICs) to work together smoothly (Cohen et al., 2007). Typically, regulatory agencies operate under their countries’ Medicines Regulatory Acts and Regulations, whereas procurement agencies operate under their countries’ Public Procurement Acts and Regulations. These independent sets of acts and regulations are not usually written to promote coordination or collaboration. In addition, many procurement agency staff in Africa lack training specifically for supply chain management of pharmaceuticals (Arney et al., 2014; Bonnifield et al., 2019).

Other factors also contribute to the challenges regulatory and procurement agencies experience in working together to ensure access to quality medical products. Overseeing markets becomes tricky if a regulator has limited capacity in terms of financial and legislative support, human resources, and other factors, as many in Africa do (Guzman et al., 2020). For example, budget and staff limitations may mean that a country’s NRA cannot effectively assess the quality of medicines procured for public healthcare facilities or accredited drug dispensing outlets (Amadi & Tsui, 2019; Ndomondo-Sigonda et al., 2017). This can deprive the national procurement agency of a valuable source of input about product quality, which is especially harmful if the agency does not have an effective quality assurance programme in place itself (Barraclough & Clark, 2012; Bonnifield et al., 2019). Another flaw in health systems is that national procurement agencies in LMICs tend to focus on price above all else (Orubu et al., 2020). This can drive quality products out of the market, as manufacturing them costs more (Bonnifield et al., 2019; Pisani, 2019; Pisani et al., 2019). Finally, global procurement programmes (e.g. the Global Fund) typically work in complement to the national procurement agency, through subsidies or direct purchase. Their quality standards for purchased products require stringent regulatory authority approval or WHO prequalification (Mace et al., 2022), but WHO prequalification only covers certain classes of medicines (e.g. for HIV, tuberculosis, malaria, and reproductive health) and global procurement programmes vary in the degree of support they provide to countries.

We call the intersection of decision-making between national regulatory and procurement agencies ‘the regulatory-procurement interface.’ Though this interface is potentially a major weakness in LMIC health systems, it is not well documented in the scientific or grey literature generated by global health researchers, practitioners, and other development partners (Bonnifield et al., 2019). The lack of research (and progress) in this area may be explained, in part, by the fact that the regulatory-procurement interface exists at the intersection of public health and commercial interests, the latter of which can unfortunately shape government policy and practice (Cohen et al., 2007; Gautier & David, 2022; Kohler et al., 2016). Weak legal systems and policies can further exacerbate these problems (Cohen et al., 2007; Kohler et al., 2016; Orubu et al., 2020). Thus, the conflicts of interest and lack of transparency inherent at this interface can be both a symptom and a cause of the challenges associated with ensuring access to quality medicines.

It is critical to address the knowledge gap around the regulatory-procurement interface for many reasons, including to attain SDG 3 and ensure a population’s access to good-quality essential medicines, but also because the global aid programmes that currently procure high-quality medicines are transitioning out of many LMICs in order to foster local sustainability (Bonnifield et al., 2019; Kohler & Ovtcharenko, 2013). In addition, many African countries are committing to the goal of universal health coverage, in which increased procurement by public institutions is expected to play an important role (Orubu et al., 2020). To learn more about the regulatory-procurement interface in Africa, in this study we used publicly available documents to analyze important characteristics of national regulatory and procurement agencies in five countries, picked to represent a diverse cross-section of sub-Saharan African nations. We adapted criteria from WHO’s *Pharmaceutical System Transparency and Accountability Assessment Tool* to identify key national policies and practices around the nexus of medicines regulation and procurement (WHO, 2018a). Here, we highlight the status of this interface in African countries, with the goal of improving access to *quality* medicines.

## Methods

### Study design

In this study, we analysed publicly available information on national procurement in five African countries. These countries were selected because they provide a range of characteristics: they cover West, East, and Southern Africa, include both small and large countries, feature low to upper-middle income levels, and two of the five have NRAs that have achieved Maturity Level 3 according to WHO’s *Global Benchmarking Tool* (Guzman et al., 2020). In presenting our results, we de-identified the five countries to avoid the appearance of criticising any particular countries and because understanding our findings is not dependent on knowing the countries’ identities. Our analysis focused on select regulatory- and procurement-related indicators based on those from an internationally recognised assessment instrument, WHO’s *Pharmaceutical System Transparency and Accountability Assessment Tool* (WHO, 2018a), as well as several indicators we created for this study. WHO’s *Pharmaceutical System Transparency and Accountability Assessment Tool* is designed to evaluate the public availability of key documentation that facilitates transparency and accountability across the pharmaceutical sector. It was developed in 2009 for the WHO Good Governance for Medicines Programme and updated in 2018 using feedback from Member States. As of 2018, it had been used in 37 countries (Martin & Ollier, 2012). To rate performance on the selected indicators, we performed a desk review of publicly available, on-line documents for each country, consistent with the current version of the tool’s recommended use.

### Indicators

We included 22 indicators from WHO’s *Pharmaceutical System Transparency and Accountability Assessment Tool* in this study. This included all available indicators for two domains: public procurement (*n* = 19) and regulation (registration; *n* = 3). For each domain, indicators fell into three types, related to standards and commitments (legislation, regulations, guidelines, standard operating procedures, and policy commitments), decisions and results (decisions of committees, declarations of interest, progress reports on policy commitments, and audit reports), and consequences and responsive actions (follow-up of investigated complaints, rulings on appeals filed, lists of corrective actions, and lists of suspended suppliers) (WHO, 2018a).

To collect additional, more granular information about the regulatory-procurement interface, we modified and added several indicators that functioned as sub-criteria for the *Pharmaceutical System Transparency and Accountability Assessment Tool*. To assess the role of quality in selecting products during the procurement process, we modified one standards-and-commitments indicator under the procurement domain, changing the wording from ‘a list/database of prices of winning contracts’ to ‘a list/database of **purchased medicines (molecule, dose, formulation, manufacturer, volume, price, timeframe)**,’ to reflect this accepted best practice for procurement (Preston et al., 2021). We also created a new indicator in the same domain: ‘published selection algorithm that includes product quality as a major factor.’ To collect additional information about the regulatory-procurement interface, we added two new registration indicators: ‘Does the public procurement agency require national registration before procurement can be done?’ (standards-and-commitments indicator) and ‘Are there information sharing activities between the drug regulatory agency and the procurement agency?’ (decisions-and-results indicator). These new and modified indicators were created under the supervision of author J.K., whose qualifications include helping to author the *Pharmaceutical System Transparency and Accountability Assessment Tool* as founding director of WHO’s Collaborating Centre for Governance, Transparency and Accountability in the Pharmaceutical Sector, and using the tool to assess over 38 countries. This process resulted in a total of 25 indicators: 20 for public procurement and 5 for regulation.

### Document identification

To assess the selected indicators, a targeted search was conducted in 2023 to identify relevant publicly accessible legislation, policy documents, and information stored on government websites. The search was conducted in parallel by two research assistants, M.C.A. and S.P. First, government institutions involved in pharmaceutical sector governance were identified for each country, and their websites were searched using indicator keywords. To complement the documents found on official government websites, Google and DuckDuckGo were employed to conduct searches of the grey literature, here defined as government documents, policy reports, institutional reports, etc. DuckDuckGo was selected because it does not provide results tailored to a searcher’s personal profile and thus can yield a broader range of primary results than Google (Briscoe et al., 2020). Variations of terminology employed in the search strategy included ‘medicines procurement’ and ‘pharmaceutical procurement.’ When no results were obtained, synonyms for the search terms just listed were iteratively tested to optimise the search and identify relevant documents.

### Assessment procedure

The same two research assistants independently scored each of the indicators using the documents they had identified in their search. After scoring the indicators, the research assistants compared their results and discussed them with author J.K. When discrepancies in ratings were identified, the group discussed the reasons for the discrepancies until consensus was reached.

Finally, assessment profiles were developed for each country based on their indicator performance to identify country-level strengths and weaknesses in the rational procurement of quality versions of pharmaceuticals. Results were then aggregated to identify commonalities between countries and their performance compared to best practices for procurement transparency and accountability. When classifying countries’ performance in various areas, if a country satisfied an indicator, it was assigned a score of 1; if it partially satisfied it, a score of 0.5; and if it did not satisfy it, a score of 0.

## Results

### Public procurement of medical products

First, we analysed the 20 indicators related to the public procurement of medical products. Ten of these indicators were related to standards and commitments (Table 1). All countries made public ‘legislation, supporting regulations, or policy on public sector procurement of medicines,’ and ‘standard operating procedures on procurement procedures.’ Thus, basic information about how the national procurement system is supposed to function is publicly available in all five countries. Performance on another key standards-and-commitments indicator was relatively high: four countries (80%) made public a ‘policy/requirement that key procurement functions and responsibilities are divided between different offices, committees, or individuals.’ Three countries (60%) made public ‘guidelines for direct purchasing,’ another straightforward aspect of procurement. Only two countries (40%) made public ‘standard operating procedures for the quantification committee (procurement committee)’ and ‘guidelines for financial audits of the procurement unit,’ with a third country partially satisfying each of these indicators. No countries had in place a ‘published selection algorithm that includes product quality as a major factor,’ information that is critical for understanding how product quality criteria are integrated into final selection decisions in relation to other factors such as price.

**Table 1.** Standards-and-commitment indicators for public procurement.

Indicator	Country A	Country B	Country C	Country D	Country E
Legislation, supporting regulations, or policy on public sector procurement of medicines	Yes	Yes	Yes	Yes	Yes
Policy/requirement that key procurement functions and responsibilities are divided between different offices, committees, or individuals?	Yes	Yes	Yes	Yes	No
Standard operating procedures for the quantification (procurement) committee?	Yes	Partial	No	No	Yes
Terms of reference for a tenders board or procurement committee(s) that is responsible for final contract decisions (adjudication)	Yes	Yes	No	Yes	Yes
Criteria on which pre-selection of suppliers for open bidding is based	Yes	Yes	Yes	Yes	No
Standard operating procedures on procurement procedures	Yes	Yes	Yes	Yes	Yes
Guidelines for direct purchasing	No	Yes	No	Yes	Yes
Published selection algorithm that includes quality as a major factor	No	No	No	No	No
Guidelines for financial audits of the procurement unit	No	Yes	No	Partial	Yes
A procurement and supply management plan	Yes	Yes	No	Partial	Yes

To assess implementation of these standards and commitments, we analysed seven decisions-and-results indicators for public procurement (Table 2). Three countries (60%) provided ‘a list/database of public sector medicines call for tenders.’ Only one country (20%) supplied ‘evidence that the procurement authority monitors supplier compliance with contracts,’ ‘summary results of financial audits of the procurement unit,’ and ‘a list of contracts for publicly procured medicines exempted from tendering.’ Although no countries made ‘a document with names and roles of appointed tender committee members publicly available,’ one country partially satisfied this indicator. Similarly, only one country partially satisfied the indicator providing for ‘a list/database of purchased medicines (molecule, dose, formulation, manufacturer, volume, price, timeframe),’ a critical practice for understanding purchases in context.

**Table 2.** Decisions-and-results indicators for public procurement.

Indicator	Country A	Country B	Country C	Country D	Country E
Evidence that the procurement authority monitors supplier compliance with contracts	No	Yes	No	No	No
List of prequalified medicines suppliers for public sector medicines tenders	Partial	Partial	Partial	Partial	Partial
A document with names and roles of appointed tender committee members publicly available	No	No	Partial	No	No
Summary results of financial audits of the procurement unit	No	Yes	No	No	No
A list/database of public sector medicines call for tenders	No	Yes	Yes	Yes	No
A list/database of purchased medicines (molecule, dose, formulation, manufacturer, volume, price, timeframe)	No	No	Partial	No	No
A list of contracts for publicly procured medicines exempted from tendering	No	No	Yes	No	No

Performance was poorest for the three consequences-and-responsive-actions indicators in this domain (Table 3). Only one country (20%) published ‘a list of suspended suppliers that have not respected their contracts,’ though another country partially met this indicator. No countries presented evidence of ‘rulings from an independent formal appeals system on appeals filed by applicants who have their bids rejected’ or ‘a list that details corrective measures enforced as identified through a financial audit.’

**Table 3.** Consequences-and-responsive-actions indicators for public procurement.

Indicator	Country A	Country B	Country C	Country D	Country E
A list that details corrective measures enforced as identified through a financial audit	No	No	No	No	No
Rulings from an independent formal appeals system on appeals filed by applicants who have their bids rejected	No	No	No	No	No
A list of suspended suppliers that have not respected their contracts	Partial	Yes	No	No	No

### Registration of medical products by national regulatory authority and characteristics of the regulatory-procurement interface

Ideally, national procurement agencies should rely on the work of their NRAs to determine whether a medical product is suitable for purchase. For this to happen, however, NRAs must be transparent about which manufacturers’ versions of products are currently registered and which products have been found problematic. To gauge whether national procurement agencies had access to this type of information, we analysed five indicators related to the registration of medical products by NRAs (Table 4).

**Table 4.** Indicators related to registration of medical products by NRAs and characteristics of the regulatory-procurement interface.

Indicator	Country A	Country B	Country C	Country D	Country E
**Standards and Commitments**					
Does the public procurement agency require national registration before procurement can be done	Yes	Yes	Yes	Yes	Yes
Criteria/procedure for the registration of medicines that have undergone prior evaluation	Yes	Yes	Yes	Yes	Yes
**Decisions and Results**					
Are there information sharing activities between the drug regulatory agency and the procurement agency?	Yes	No	No	No	No
A list/database of all pharmaceutical products registered in the country updated at least annually (and information categories available, e.g. generic names, dosage form, strength, marketing authorisation holder, etc.)	Yes	Yes	Yes	Yes	Yes
**Consequences and Responsive Actions**					
A list/database of products that had their market authorisation suspended or revoked	No	No	No	No	No

All five countries offered evidence that ‘the public procurement agency requires national registration before procurement can be done’ and provided a ‘list/database of all pharmaceutical products registered in the country updated at least annually (and information categories available, e.g. generic names, dosage form, strength, marketing authorization holder, etc.).’ Based on this information, the national procurement agencies in the five target countries should be able to, at least theoretically, base initial eligibility decisions on whether a particular manufacturer’s version of a medical product has been authorised for marketing by their NRAs. In addition, all five countries had in place ‘criteria/procedure for the registration of medicines that have undergone prior evaluation.’ This signifies that each country can practice regulatory reliance, as they have in place processes to quickly grant marketing authorisations to medical products previously reviewed and authorised by trusted reference agencies, such as stringent regulatory authorities, WHO-listed authorities, and the WHO Prequalification Programme. This can bolster scrutiny of the quality of specific versions of products, particularly where national regulatory capacity is limited. Only one country (20%) presented evidence of ‘information sharing activities between the drug regulatory agency and the procurement agency.’ None of the countries’ NRAs had in place ‘a list/database of products that had their market authorization suspended or revoked.’ The absence of this information could make it difficult for national procurement agencies to bar problematic products (and their suppliers) from future tenders.

### Summary of overall procurement landscape

**Figure 1.**
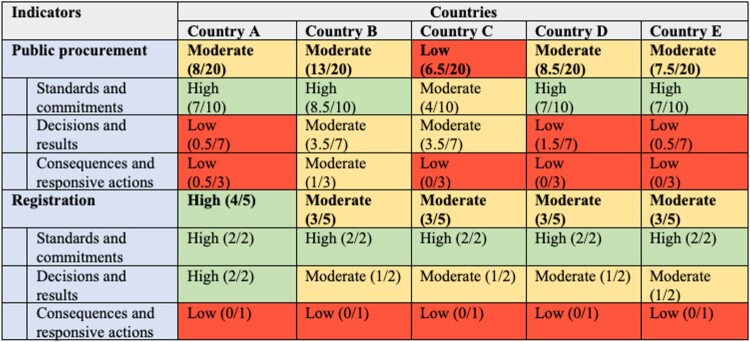
Overall performance landscape for indicators related to the interface of the registration and procurement of medical products. Classification of high/moderate/low follows the instructions in WHO’s *Pharmaceutical System Transparency and Accountability Assessment Tool*: green indicates a country met >66% of indicators in a certain area (high); yellow, 33%-66% (moderate); and red <33% (low) (WHO, 2018a). If a country satisfied an indicator, it was assigned a score of 1; if it partially satisfied it, a score of 0.5; and if it did not satisfy it, a score of 0.

The countries’ aggregate results (Figure 1) show that though performance was moderate for the procurement and registration indicators overall, it was uneven across the three classes of indicators. Whereas performance on standards-and-commitments indicators was generally high, performance on decisions-and-results indicators was lower, and performance on consequences-and-responsive actions indicators was the worst.

## Discussion

In this study, we used publicly accessible documents to analyze the regulatory-procurement interface in five African countries. This sample of five countries represents some of the better-functioning health systems in sub-Saharan Africa. Even so, we identified important opportunities to strengthen the regulatory-procurement interface and enhance access to quality medical products.

Legal and policy frameworks enabling best practices in procurement (as revealed by the standards-and-commitments indicators) were often in place in these countries, but implementation and enforcement of these best practices (as represented by the decisions-and-results and consequences-and-responsive-actions indicators) appear to be key areas for strengthening. This is not to say that best practices were always present for standards-and-commitments indicators. For example, only three of the five countries had public guidelines for the direct purchasing of medical products. If many medical products receive waivers, or are directly purchased without competition, the procurement process presented to the public may be regularly circumvented, creating quality vulnerabilities (Cohen et al., 2007).

However, in terms of decisions-and-results indicators, only one country provided a list of contracts for publicly procured medicines exempted from tendering, limiting transparency on when exceptions to normal procurement processes are granted. In terms of consequences-and-responsive-actions indicators, no countries published a list/database of products that had their market authorisation suspended or revoked, and only one country provided a full list of suspended suppliers that have not respected their contracts. Without these lists, it is difficult for the government to act against, and for the public to discern, those actors that are flouting the public procurement system (Cohen et al., 2007; Kohler & Ovtcharenko, 2013).

Despite the importance of prioritising product quality when making procurement decisions, we documented a lack of publicly available information related to the role that product quality plays in selecting medical products. We found no published algorithms for how quality criteria factor into the selection of products for procurement in *any* of the countries. Without such information, it is not possible to understand how quality is weighed when making procurement decisions. If quality is not favoured and price is the primary criterion for selection, then the manufacturers of high-quality products will have little incentive to submit them to a country, as they are bound to lose bids to lower-cost/lower-quality medical products (Bonnifield et al., 2019; Pisani, 2019; Pisani et al., 2019).

Moreover, none of the five countries appear to publish selection decisions by purchased product, including information on molecule, dose, formulation, manufacturer, volume, price, timeframe, etc., although one country made available a list that partially met these criteria. Without detailed information on selected products, it is difficult for citizens to assess whether basic quality standards are being met and hold their governments to account. Manufacturer information is important because, for example, it can be used to search for a history of major regulatory infractions (e.g. manufacturers linked to diethylene glycol poisoning). In addition, if a manufacturer only holds authorisations in countries with little regulation, this could indicate an intentional strategy to evade rigorous scrutiny, implicating product quality (though there are also reputable manufacturers who specialise in serving LMIC markets) (Preston et al., 2012, 2021). Information on molecule, dose, formulation, and place of manufacture is important because it can be used to check for approval by stringent regulatory authorities, WHO-listed authorities, and/or WHO’s Prequalification Programme. If a manufacturer makes a product whose molecule, dose, and formulation appear to be approved by one of these entities, but the company does not acknowledge this approval when applying for marketing authorisation in an LMIC, it raises questions about tiered manufacturing. Tiered manufacturing occurs when a manufacturer sells the same molecule, dose, and formulation of a medicine to different markets but makes the product according to different standards and in different facilities depending on the rigour of oversight in the receiving market (Caudron et al., 2008; Preston et al., 2021). When tiered manufacturing occurs, countries with less regulatory oversight are more likely to receive lower-quality versions of a product.

Finally, price information is critical because it can inform inferences about product quality. If a country’s procurement agency consistently purchases products at or below the low end of the price-per-unit spectrum on international pricing indices, for example, it could imply lower product quality. Faced with limited budgets and pressure to minimise expenditures, procurement agencies in LMICs understandably put a heavy emphasis on price over other factors. Unfortunately, this approach can drive quality products out of the market, as manufacturers and distributors often maintain profits by cutting production costs, including by using cheaper ingredients or skipping quality assurance steps (which can lead to tiered manufacturing) (Pisani, 2019; Pisani et al., 2019).

We also documented a disconnect between the work of countries’ NRAs and their procurement agencies, which is likely to impede procurement agencies’ ability to make informed decisions about product quality (Cohen et al., 2007). In many African countries, procurement is a multistep process that begins with a technical team, led by NRA staff, that selects the products to be procured (typically based on WHO’s Essential Medicines List and lists of prequalified products); continues with a procurement unit, which collects commercial information from bidders; and ends with a commercial committee that processes purchasing decisions. This separation of duties creates ample opportunity for purchasing decisions to become disconnected from medical product regulations, as well as NRA approvals and withdrawals (Boche et al., 2022; Pisani et al., 2019). We found clear descriptions of information sharing activities between NRAs and procurement agencies in only one of the five countries. Furthermore, we found none of the five NRAs published a list of products that had their marketing authorisation suspended or revoked. This information is essential for helping procurement agencies avoid purchasing SF medicines, and publishing such a list would also keep the public informed about the quality of different medicines and manufacturers. Formalising the mechanisms through which information flows between NRAs and procurement agencies (e.g. via information sharing agreements) could help ensure procurement agencies have the information they need to make optimal purchasing decisions.

Based on the countries’ performance on the indicators assessed in this study, we believe one of the most important next steps for improving the effectiveness and transparency of national procurement is for regulatory and procurement agencies to jointly develop and publish detailed quality selection criteria, based on countries’ medicines regulation frameworks. Another important step is for procurement agencies to publish up-to-date lists of the products they have purchased, with the key product information outlined in this article. Experts have been calling for increased visibility of procurement data, including price and product quality information, for years (Chalkidou et al., 2020). One way to promote the availability of such data would be to either use existing data collection tools or develop new ones to encourage countries to collect and share this information in a standardised way. Transparent e-procurement systems are one promising approach to improving visibility into countries’ procurement processes and decisions.

The global health community could support procurement of quality medicines by adding relevant indicators to prominent tools and goals. For example, new indicators requiring detailed procurement data could be added to WHO’s *Pharmaceutical System Transparency and Accountability Assessment Tool* (WHO, 2018a), and new indicators assessing the maturity of a country’s regulatory-procurement interface could be added to WHO’s *Global Benchmarking Tool* (Guzman et al., 2020). Making product quality a key performance indicator in important initiatives such as the SDGs could help draw the world’s attention to this area, bringing with it funding and other types of support.

Furthermore, the global health community (WHO, World Bank, etc.) could support best practices for the regulatory-procurement interface by mirroring the specialized support it has offered to strengthen NRAs and regional regulatory bodies around the world. Up-to-date global guidance is needed on best practices for procurement, including how to select quality medical products and implement best practices at the local, national, and regional level (Bonnifield et al., 2019). Updated and expanded guidance might address (1) selecting and monitoring for product quality when regulatory capacity is limited; (2) leveraging reliance on global and regional systems and structures to assure product quality; and (3) the laws, policies, and practices regulatory and procurement agencies need to better work together to ensure quality. It is equally important, of course, that regulatory and procurement agencies receive the resources and political support to enforce these laws and regulations (Bonnifield et al., 2019; Kohler et al., 2016).

The global health community could also foster more professional interaction and training at the regulatory-procurement interface, as many staff members at national procurement agencies in Africa currently lack expertise in supply chain management specifically for pharmaceuticals (Boche et al., 2022; Bonnifield et al., 2019). One idea for working toward this goal is setting up a community of practice dedicated to country-level procurement of quality medicines (Bonnifield et al., 2019; Chalkidou et al., 2020). Establishing fora for procurement and regulatory professionals (including chief pharmacists) to work together could also be highly valuable (Caribbean Pharmaceutical Policy, 2013). Additionally, procurement professionals would benefit from dedicated training or courses on quality selection in medical product procurement, perhaps modelled on the World Bank’s successful Health Systems Flagship Program and Course (Bonnifield et al., 2019). Finally, mentoring and exchanges between more and less mature programmes have worked well for NRA capacity building and could do the same for procurement (Bonnifield et al., 2019; Mashingia et al., 2020; Ndomondo-Sigonda et al., 2023).

This study has several important limitations. First, as recommended by the instructions for WHO’s *Pharmaceutical System Transparency and Accountability Assessment Tool*, we relied on documents readily available on the web to assess each country’s performance on the selected indicators. As a result, we may have failed to identify relevant information in documents that can only be accessed through in-person archives, upon request from the relevant institutions, or through key informant interviews. In addition, some procurement agencies and regulators may be satisfying an indicator without publicly documenting their work in that area. Because national procurement and regulatory agencies work on behalf of the government, we argue information about these critical indicators should be available to the public – and that lack of public information about these actions is probably a sign they are not being performed. Second, we analysed the regulatory-procurement interface in only five countries, many of which had among the stronger health systems in sub-Saharan Africa. This may have led us to underestimate the magnitude of regulatory-procurement disconnects. In the future, it will be important to assess procurement and registration indicators in more countries. Third, there has been a trend toward decentralisation in public procurement in African countries (Arney et al., 2014). To the extent that different procurement foci with different procedures exist in the same country, it may be difficult for a government to present a unified description of the procurement procedures in place. Finally, many individuals in LMICs rely on medicines not purchased by national procurement agencies (Kettler et al., 2020), such as those purchased in the private sector, and our study does not address this sphere. Though private sector procurement should ostensibly obey the relevant NRA’s decisions, regulatory oversight is challenging. Lapses in this system are another major source of SF medicines in LMICs (McManus & Naughton, 2020) and also need critical attention.

## Conclusion

This study represents a snapshot of the regulatory-procurement interface in five African countries, obtained using a standardised tool and publicly available, web-based, English language documents. Addressing the common challenges identified in this study is growing increasingly urgent, particularly given the current imbalance in SF product levels in Africa versus the rest of the world. Other factors, such as the move toward universal health coverage in Africa and donor programmes transitioning to country ownership, also make swift action critical. Considering the common challenges documented in this study, we hope our findings can help inform the conversation about implementing and enforcing best practices at the regulatory-procurement interface (e.g. greater transparency around quality selection and purchased medicines; information sharing about SF products authorised or procured), with the goal of improving access to quality versions of medicines in Africa and around the world.
